# The effect of preoperative use of anticoagulants on the hemostatic effect of intravenous application of tranexamic acid in PLIF: a case control study

**DOI:** 10.1038/s41598-024-60440-9

**Published:** 2024-05-14

**Authors:** Shenshen Hao, Binbin Li, Shiying Luo, Shengli Dong, Shuai Liu, Hongke Li, Xinhao Cao

**Affiliations:** 1 Department of Spine and Bone Oncology, General Hospital of Pingmei Shenma Medical Group, Pingdingshan City, Henan Province China; 2 Department of Rehabilitation Medicine, Haikou Hospital Affiliated to Xiangya Medical College of Central South University, Haikou City, Hainan Province China; 3 Office of the Ombudsman, Pingdingshan University, Pingdingshan City, Henan Province China; 4 Emergency Department, Xi’an Honghui Hospital, No. 555, Youyi East Road, Nanshaomen, Beilin District, Xi’an City, 710000 Shaanxi Province China

**Keywords:** Lumbar degenerative disease, Posterior lumbar interbody fusion, Tranexamic acid, Anticoagulants, Coagulation indicators, Hemostatic effect, Diseases, Medical research

## Abstract

Intravenous application of tranexamic acid (TXA) in posterior lumbar interbody fusion (PLIF) can effectively reduce blood loss without affecting coagulation function. However, it has not been reported whether preoperative use of anticoagulants may affect the efficacy of TXA in PLIF. The purpose of this study is to observe the effect of preoperative use of anticoagulants on coagulation indicators and blood loss after PLIF receiving intravenous unit dose TXA. A retrospective analysis was conducted on data from 53 patients with PLIF between 2020.11 and 2022.9, who received intravenous application of a unit dose of TXA (1 g/100 mL) 15 min before the skin incision after general anesthesia. Those who used anticoagulants within one week before surgery were recorded as the observation group, while those who did not use anticoagulants were recorded as the control group. The main observation indicators include surgical time, intraoperative blood loss, postoperative drainage volume, blood transfusion, and red blood cell (RBC), hemoglobin (HB), and hematocrit (HCT) measured on the 1st, 4th, 7th, and last-test postoperative days. Secondary observation indicators included postoperative incision healing, deep vein thrombosis of lower limbs, postoperative hospital stay, and activated partial thrombin time (APTT), prothrombin time (PT), thrombin time (TT), fibrinogen (FIB), and platelets (PLT) on the 1st and 4th days after surgery. The operation was successfully completed in both groups, the incision healed well after operation, and no lower limb deep vein thrombosis occurred. There was no significant difference in surgical time, intraoperative blood loss, postoperative drainage volume, and blood transfusion between the two groups (*p* > 0.05). There was no significant difference in the RBC, HB, and HCT measured on the 1st, 4th, 7th, and last-test postoperative days between the two groups (*p* > 0.05). There was no statistically significant difference in APTT, PT, TT, FIB and PLT between the two groups on the 1st and 4th postoperative days (*p* > 0.05). There was no significant difference in postoperative hospital stay between the two groups (*p* > 0.05). The use of anticoagulants within one week before surgery does not affect the hemostatic effect of intravenous unit dose TXA in PLIF.

## Introduction

Posterior lumbar interbody fusion (PLIF) is a common and effective operation for the treatment of lumbar disc herniation (LDH), lumbar spinal stenosis (LSS), lumbar spondylolisthesis (LS) and other lumbar diseases^1,2^. However, it faces the challenge of massive perioperative blood loss^3^. Tranexamic acid (TXA) is a synthetic derivative of lysine, a synthetic anti fibrinolytic drug that could reduce surgical bleeding by inhibiting fibrinolysis and stabilizing blood clots^4^. Many studies have shown that intravenous TXA can effectively reduce perioperative blood loss in PLIF^5–8^. Meanwhile, in our previous studies, we confirmed that preoperative intravenous unit dose TXA in PLIF can safely and effectively reduce intraoperative and postoperative blood loss without affecting postoperative coagulation function indicators^9,10^.

However, in clinical practice, we have found such a phenomenon. Some patients who require surgery have a history of using anticoagulants for treatment before hospitalization. Therefore, it is inevitable to use anticoagulants before surgery to maintain treatment. In theory, the use of anticoagulants before surgery may increase perioperative blood loss. However, it is not yet known whether the hemostatic effect of TXA in PLIF may be affected by preoperative anticoagulant drugs. Therefore, this study mainly explores the following two issues. One is the safety of using anticoagulants within one week before surgery in PLIF receiving intravenous unit dose TXA. The other is the effect of using anticoagulants within one week before surgery on the hemostatic effect of TXA in PLIF.

## Method

### Study design

This study was a retrospective, single center case control study. It was approved by NO.2021004, from the Ethics Committee of the General Hospital of Pingmei Shenma Medical Group. This study had been performed in accordance with the Declaration of Helsinki. The medical records were collected in the hospital, and the time range was 2020.11–2022.9. The inclusion criteria include: *a*. preoperative diagnosis of LDH, LSS, and LS, receiving standard PLIF treatment, *b*. general anesthesia, *c*. surgical segments ranging from 1 to 3, *d*. age range from 50 to 80 years, *e*. intravenous application of a unit dose of TXA (1 g/100 mL) (containing 100 mL normal saline and 1 g TXA) 15 min before the skin incision after anesthesia. Exclusion criteria include: *a*. pre-operative history of blood disease, *b*. preoperative history of deep vein thrombosis (DVT), *c*. lumbar surgery history, *d*. history of diabetes, *e*. intraoperative cerebrospinal fluid leakage or dural injury. Finally, 53 eligible cases were included. Among them, there are 21 males and 32 females, with an average age of (63.7 ± 8.5) years. The criteria for grouping were based on whether anticoagulants, such as low molecular weight heparin (LMWH) or indoprofen tablets, were used within one week before surgery. 25 cases of preoperative use of anticoagulants were recorded as the observation group, 28 cases were not applied and recorded as the control group. The intraoperative and postoperative measures of PLIF were similar. After surgery, two drainage tubes were placed and removed when the drainage flow rate was less than 50 mL/24 h. Postoperative routine use of antibiotics to prevent infection, corticosteroids to reduce spinal cord stress response, dehydration drugs to reduce edema response, non steroidal drugs to reduce pain, and LMWH drugs or indobufen tablets to prevent DVT.

### Outcome indicators

Preoperative patient information was collected as baseline data. They included age, gender, body mass index (BMI), disease type, surgical segment, coexisting hypertension, prothrombin time (PT), activated partial thrombin time (APTT), thrombin time (TT), fibrinogen (FIB), platelets (PLT), hemoglobin (HB), red blood cell (RBC), and hematocrit (HCT).

The main observation indicators include surgical time, intraoperative blood loss, postoperative drainage volume, blood transfusion, and RBC, HB, and HCT measured on the 1st, 4th, 7th, and last-test postoperative days. The trend of changes in HB, RBC, and HCT during the perioperative period, represented by the median, was plotted using an Excel table.

Secondary observation indicators included postoperative incision healing, DVT of lower limbs, postoperative hospital stay, APTT, PT, TT, FIB, and PLT on the 1st and 4th postoperative days. The trend of changes in APTT, PT, TT, FIB, and PLT during the perioperative period was represented by the median, and a column chart was drawn using an Excel table.

### Statistical methods

Data analysis was performed via SPSS statistical software (version 22.0). The econometric data which met the criteria was represented by mean ± standard deviation, and t-test was used for inter groups comparison. Non-conformities were represented by M [P25; P75], and comparisons between groups were conducted using Mann–Whitney U non-parametric tests. The counting data was expressed in the number of cases, and the Chi-squared test was used to compare between groups. The comparison of RBC, HB, HCT, APTT, PT, TT, FIB, and PLT between two groups measured on the 1st, 4th, 7th, and last-test postoperative days was conducted using a linear mixed model. *P* < 0.05 was considered statistically significant.

### Ethics approval and consent to participate

This study was approved by the Ethics Committee of the General Hospital of Pingmei Shenma Medical Group, and the reference number is 2021004. This study had been performed in accordance with the Declaration of Helsinki. All authors confirmed that informed consent was obtained from all subjects.

## Results

### The comparison results of baseline data between the two groups

There was no significant differences in baseline data, concluding age, gender, BMI, disease type, surgical segment, coexisting hypertension, PT, APTT, TT, FIB, PLT, HB, RBC and HCT, between the two groups (*p* > 0.05), as shown in Table 1.

**Table 1:** Comparison of baseline data between the two groups.

Groups	Observation group(n = 25)	Control group(n = 28)	t/χ^2^/Z	*p*
Age, year	65.7 ± 8.5	61.9 ± 8.2	1.651	0.105
Gender, n			1.149	0.284
Male	8	13		
Female	17	15		
BMI, kg/m^2^	24.460 ± 3.402	23.373 ± 2.469	1.341	0.186
Disease type, n			0.358	0.836
LDH	4	3		
LSS	14	16		
LS	7	9		
Surgical segment, n			− 0.608	0.543
One	9	14		
Two	14	10		
Three	2	4		
Coexisting hypertension, n			0.759	0.384
Yes	8	6		
No	17	22		
APTT, s	31.176 ± 2.946	31.561 ± 2.526	− 0.512	0.611
PT, s	11.300 ± 0.785	11.432 ± 0.812	− 0.601	0.551
TT, s	14.50 [13.70; 15.10]	15.15 [13.90; 15.66]	− 1.418	0.156
FIB, g/L	3.023 ± 0.507	2.915 ± 0.611	0.699	0.488
PLT, 10^9^/L	220 [192; 254]	203 [176; 238]	− 1.265	0.206
HB, g/L	132.880 ± 15.425	138.143 ± 8.793	− 1.502	0.142
RBC, 10^12^/L	4.218 ± 0.465	4.290 ± 0.399	− 0.604	0.549
HCT, L/L	0.387 ± 0.043	0.403 ± 0.025	− 1.663	0.105

### The comparison of main observation indicators between the two groups

**Figure 1:**
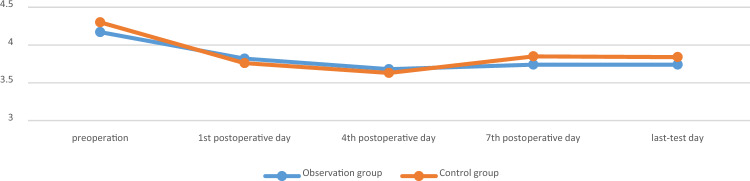
The changes of RBC of the two groups, 10^12^/L.

**Figure 2:**
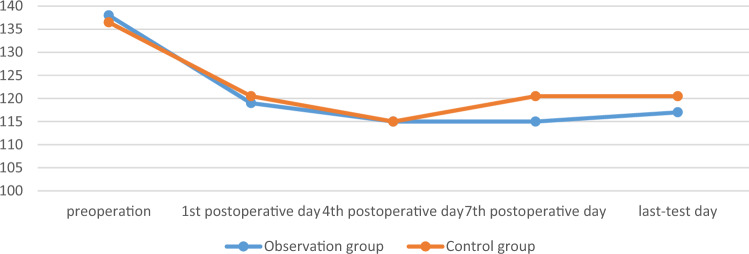
The changes of HB of the two groups, g/L.

**Figure 3:**
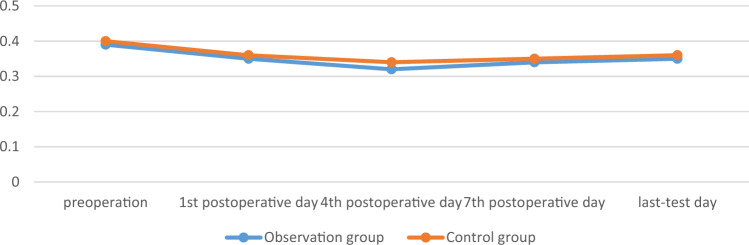
The changes of HCT of the two groups, L/L.

There was no significant difference in surgical time, intraoperative blood loss, postoperative drainage volume, and blood transfusion between the two groups (*p* > 0.05). There was no significant difference in the RBC, HB, and HCT measured on the 1st, 4th, 7th, and last-test postoperative days between the two groups (*p* > 0.05). The comparison was shown in Tables 2 and 3, Figs. 1, 2 and 3.

**Table 2:** Comparison of main observation indicators between the two groups.

Groups	Observation group (n = 25)	Control group (n = 28)	t/χ^2^/Z	*p*
Operation time, min	180 [165; 230]	166 [135; 205]	− 1.623	0.105
Intraoperative blood loss, mL	300 [300; 500]	300 [200; 500]	− 0.383	0.702
Postoperative drainage volume, mL	235 [200; 300]	225 [195; 270]	− 0.805	0.421
Blood transfusion, n			0.889	0.346
Yes	5	3		
No	20	25		
Postoperative hospital stay, day	15 [10; 20]	13 [10; 17]	− 0.822	0.411

**Table 3:** Comparison of main observation indicators between the two groups.

Groups	Observation group(n = 25)	Control group(n = 28)	F	*p*
HB, g/L				
Sphericity test				0.039
Group			1.47	0.226
Time			4.39	0.008
Group*measured-time			1.89	0.143
1st day	116.080 ± 15.226	122.679 ± 14.048		0.107
4th day	113.960 ± 18.492	117.643 ± 15.191		0.430
7th day	118.240 ± 16.236	120.143 ± 13.080		0.639
Last-test day	120.240 ± 12.115	120.821 ± 13.185		0.868
RBC, 10^12^/L				
Sphericity test				< 0.001
Group			0.53	0.713
Time			1.41	0.252
Group*measured-time			0.72	0.544
1st day	3.754 ± 0.489	3.826 ± 0.479		0.595
4th day	3.68 [3.31; 4.16]	3.63 [0.33;4.14]		0.726
7th day	3.786 ± 0.512	3.772 ± 0.474		0.919
Last-test day	3.856 ± 0.389	3.830 ± 0.470		0.824
HCT, L/L				
Sphericity test				< 0.001
Group			1.61	0.188
Time			2.10	0.112
Group*measured-time			0.72	0.544
1st day	0.340 ± 0.042	0.357 ± 0.042		0.142
4th day	0.32 [0.31; 0.37]	0.34 [0.31; 0.38]		0.308
7th day	0.345 ± 0.046	0.349 ± 0.039		0.727
Last-test day	0.354 ± 0.035	0.355 ± 0.039		0.923

### The comparison of secondary observation indicators between the two groups

**Figure 4:**
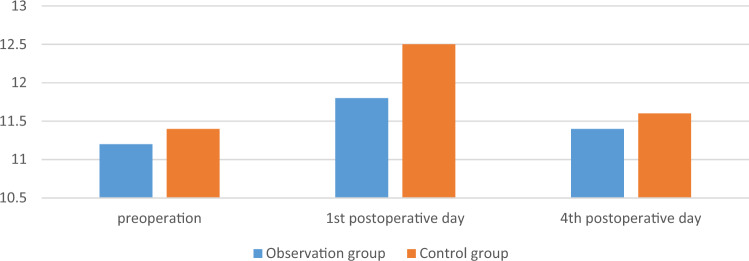
The changes of PT of the two groups, s.

**Figure 5:**
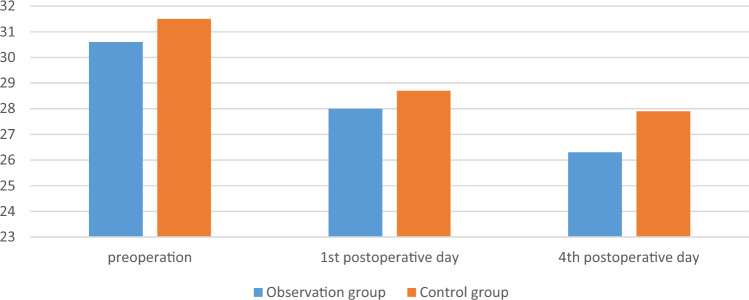
The changes of APTT of the two groups, s.

**Figure 6:**
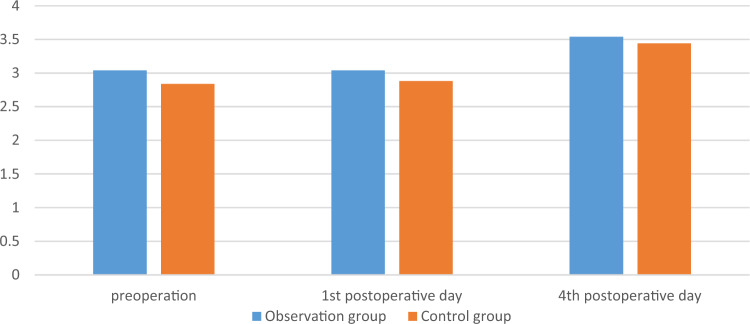
The changes of FIB of the two groups, g/L.

**Figure 7:**
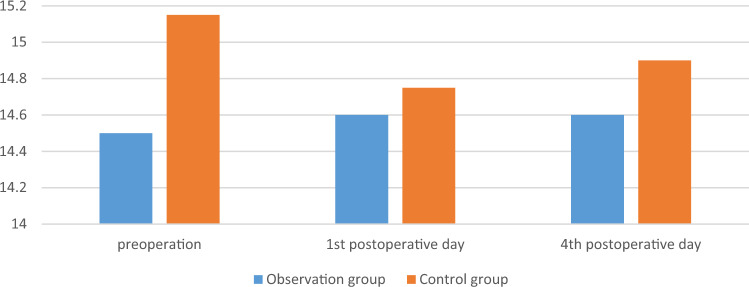
The changes of TT of the two groups, s.

**Figure 8:**
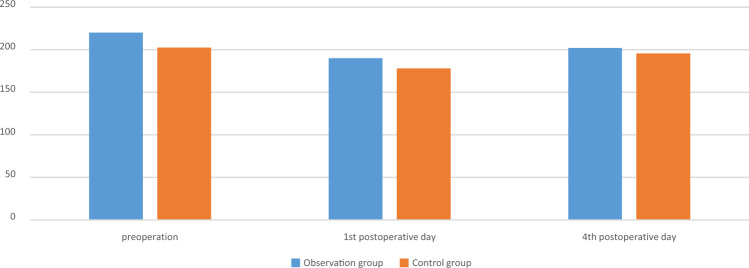
The changes of PLT of the two groups, 10^9^/L.

The surgery was successfully completed in both groups of patients, with good postoperative incision healing and no occurrence of DVT. There was no significant difference in APTT, PT, TT, FIB and PLT between the two groups on the 1st and 4th postoperative days (*p* > 0.05). The comparison was shown in Table 4, Figs. 4, 5, 6, 7 and 8.

**Table 4:** Comparison of secondary observation indicators between the two groups.

Groups	Observation group (n = 25)	Control group (n = 28)	F	*p*
PT, s				
Group			1.23	0.272
Time			0.35	0.557
Group*measured-time			0.77	0.385
1st day	11.904 ± 0.858	12.325 ± 0.912		0.091
4th day	11.4 [11.0; 11.9]	11.6 [11.0; 12.6]		0.324
APTT, s				
Group			1.16	0.286
Time			10.17	0.002
Group*measured-time			1.92	0.172
1st day	28.0 [27.3; 29.5]	28.7 [28.1; 30.2]		0.626
4th day	26.3 [25.7; 28.5]	27.9 [26.0; 29.1]		0.158
TT, s				
Group			0.71	0.405
Time			2.24	0.141
Group*measured-time			2.12	0.151
1st day	14.459 ± 1.004	14.468 ± 1.339		0.979
4th day	14.6 [13.9; 15.2]	14.9 [14.1; 15.5]		0.163
FIB, g/L				
Group			0.03	0.856
Time			11.98	0.001
Group*measured-time			0.59	0.447
1st day	3.04 [2.85; 3.46]	2.88 [2.55; 3.22]		0.506
4th day	3.439 ± 0.734	3.500 ± 0.764		0.769
PLT, 10^9^/L				
Group			0.11	0.741
Time			5.03	0.029
Group*measured-time			0.02	0.876
1st day	190 [174; 225]	178 [151; 231]		0.778
4th day	202 [182; 218]	196 [156; 234]		0.720

## Discussion

The safety and feasible of PLIF have been recognized by spine surgeon and patients. There are four main reasons for bleeding in PLIF^11^. Firstly, the muscles in the surgical area of the lower back are very developed, so it is easy to bleed when peeling off the paraspinal muscles and soft tissues during surgery. Secondly, after decompression through laminectomy, the cancellous bone surface is prone to bleeding. Thirdly, when decompression enters the vertebral canal, it is easy to damage the intervertebral venous plexus and cause bleeding. Fourthly, when removing the intervertebral disc, it is necessary to scratch the upper and lower endplates, which is prone to bleeding. In addition, during surgery, a decrease in the number of coagulation factors and hyperfibrinolysis in patients can lead to significant intraoperative bleeding^12^. On the one hand, spine surgeon should consider the effect of PLIF, and on the other hand, consider how to reduce perioperative blood loss^13^. Many scholars have studied that intravenous TXA can safely and effectively reduce perioperative blood loss in PILF patients^5–8^. There are various options for using TXA during the perioperative period of spinal surgery, such as oral medication, local medication, intravenous medication, etc. ^14,15^. However, the most commonly used method is intravenous administration^16^. In terms of medication timing, Yu et al.^17^ suggested that during posterior lumbar surgery, intravenous administration of TXA 15 min before skin incision can reduce perioperative bleeding. This is because after about 15 min of intravenous application of TXA, it can reach and accumulate in the surgical area to effectively exert its hemostatic effect^18^. Therefore, in this study, TXA was chosen to be administered intravenously 15 min before PLIF. This usage is also one of the recommended methods in the Chinese Expert Consensus^19^.

In clinical practice, there are cases where some patients use anticoagulants before surgery. Vitamin K antagonists represented by Warfarin and antiplatelet drugs represented by aspirin will increase the risk of intraoperative bleeding, so these drugs are not used during perioperative preventive anticoagulation^20^. Heparins are widely used anticoagulants in clinical practice, mainly including ordinary heparin and LMWH. The relative molecular weight of LMWH is smaller than that of ordinary heparin, and it can only bind to antithrombin factor IIIa and cannot bind to factor IIa, thus possessing anti factor Xa effects^21^. LMWH can inhibit the activation of thrombin, thereby playing an anti-thrombotic role^22^. Meanwhile, compared to ordinary heparin, LMWH has a longer half-life, better bioavailability, better subcutaneous absorption, more stable pharmacokinetics, fewer adverse reactions such as bleeding, allergic reactions, and heparin induced thrombocytopenia, making it widely used in clinical practice^23^. Some studies have proved that the anticoagulant efficacy of factor Xa inhibitor represented by Rivaroxaban is consistent with LMWH, and does not increase the risk of serious bleeding, but it is currently mainly used after hip and knee joint replacement^24,25^. Indobufen, as a new generation of antiplatelet drugs, has good anticoagulant effects^26^. It can effectively inhibit the activity of platelet cyclooxygenase 1 , which is similar to the biochemical, functional and clinical effects of standard dose aspirin^27^. Indobufen tablets are oral medications and are more suitable for patients who are unwilling to receive subcutaneous injection of LMWH. Therefore, the anticoagulants used by the patients in this study were LMWH and indobufen tablets. In theory, for such patients, intraoperative and postoperative bleeding may increase.

However, the results of this study found that the intraoperative blood loss, postoperative drainage volume, and blood transfusion rate were similar in both groups. This indicates that for PLIF, TXA has a similar hemostatic effect in patients who use anticoagulants before surgery compared to those who do not. In order to further observe the possible correlation between the two types of drugs, namely synergistic or antagonistic effects. This study continuously observed RBC, HB, and HCT at different stages after surgery, and found no significant difference between the two groups, and both groups remained relatively stable. On the other hand, this suggests that the hemostatic effect of TXA in PLIF is not affected by the use of anticoagulants before surgery. It is worth noting that the PLIF surgical technique is already very mature, and the perioperative treatment measures are also very standardized. Moreover, both groups of patients in this study adopted similar treatment plans. These may be some of the possible reasons why the above indicators are similar. Therefore, further research is needed on the possible interrelationships between anticoagulants and TXA.

At the same time, patients receiving PLIF are mostly elderly people who require bed rest after surgery, and there are high-risk factors for thrombosis^28^. TXA, as an anti fibrotic hemostatic drug, may theoretically increase the risk of DVT^29,30^. The use of anticoagulants before surgery may affect postoperative coagulation function and recovery process. It is not yet known whether the simultaneous use of two types of drugs in a short period of time will have an impact on the safety of surgery. As is well known, surgical safety is a prerequisite for PLIF. Therefore, this study investigated the safety of surgery. In this study, not only were the surgeries completed safely in both groups, but there were no adverse events after the surgery, and the postoperative hospital stay was similar. Moreover, postoperative coagulation function indicators (APTT, PT, TT, FIB) and PLT were also similar. These indicate that preoperative use of anticoagulant drugs will not have adverse effects on the perioperative safety and postoperative recovery cycle of PLIF patients who have received intravenous TXA. There are two possible reasons for this phenomenon. Firstly, the fibrinolytic system and coagulation system are two independent systems, and there may be no related interference between anti-fibrinolytic and anticoagulant systems. Secondly, the sample size of this study is small and may have some bias. However, overall, TXA is safe and feasible for PLIF application in preoperative anticoagulant patients.

## Conclusion

Through this study, two issues mentioned at the beginning were resolved. Firstly, it is safe and feasible to use anticoagulants within one week before surgery in PLIF with intravenous TXA. Secondly, the use of anticoagulants within one week before surgery did not affect the hemostatic effect of intravenous TXA on PLIF. However, this study is a single center, small sample retrospective medical record control study, and the reliability of its conclusions may inevitably be affected to some extent. Further research is needed to support the application of TXA in preoperative anticoagulation patients.

## Data Availability

The datasets used and analyzed during the current study are available from the corresponding author upon reasonable request.

## References

[1] Nagata K, Yoshimura N, Hashizume H, Ishimoto Y, Muraki S, Yamada H, Oka H, Kawaguchi H, Akune T, Tanaka S (2017). The prevalence of tandem spinal stenosis and its characteristics in a population-based MRI study: The Wakayama Spine Study. Eur. Spine J..

[2] Martin BI, Mirza SK, Spina N, Spiker WR, Lawrence B, Brodke DS (2019). Trends in lumbar fusion procedure rates and associated hospital costs for degenerative spinal diseases in the United States, 2004 to 2015. Spine.

[3] Smilowitz NR, Oberweis BS, Nukala S, Rosenberg A, Zhao S, Xu J, Stuchin S, Iorio R, Errico T, Radford MJ (2016). Association between anemia, bleeding, and transfusion with long-term mortality following noncardiac surgery. Am. J. Med..

[4] Adigweme O, Lee G-C (2017). Tranexamic acid: The new gold standard?. Tech. Orthop..

[5] Todeschini AB, Uribe AA, Echeverria-Villalobos M, Fiorda-Diaz J, Abdel-Rasoul M, McGahan BG, Grossbach AJ, Viljoen S, Bergese SD (2020). Efficacy of intravenous tranexamic acid in reducing perioperative blood loss and blood product transfusion requirements in patients undergoing multilevel thoracic and lumbar spinal surgeries: A Retrospective study. Front. Pharmacol..

[6] Yan L, Yang H, Jiang H, Yu M, Tan J, Su T, Xu G (2021). Impact of the tranexamic acid on bleeding amount of surgical patient with degenerative spinal disease: A randomized blinded study. Front. Surg..

[7] Wang F, Wang J, Nan L, Zhou S, Liu Y, Cai T, Wang S, Chen D, Feng X, Zhang L (2019). The effectiveness and safety of tranexamic acid in posterior lumbar interbody fusion: A placebo-controlled randomized study. Chin. J. Spine Spinal Cord.

[8] Li J, Wang L, Bai T, Liu Y, Huang Y (2020). Combined use of intravenous and topical tranexamic acid efficiently reduces blood loss in patients aged over 60 operated with a 2-level lumbar fusion. J. Orthop. Surg. Res..

[9] Hao S, Wang X, Yue Z, Zhang R, Wang P, Meng S, Liu S, Li H, Dong S (2022). RBC, HB, HCT, CRP, and ESR at different postoperative periods after the application of intravenous unit dose transient acid in PLIF: A case control study. Front. Surg..

[10] Hao S, Li H, Liu S, Meng S, Zhang X, Wang L, Yang H, Zhang L, Dong S (2023). The effect of intravenous unit-dose tranexamic acid on visible and hidden blood loss in posterior lumbar interbody fusion: A randomized clinical trial. Sci. Rep..

[11] Mikhail C, Pennington Z, Arnold PM, Brodke DS, Chapman JR, Chutkan N, Daubs MD, DeVine JG, Fehlings MG, Gelb DE (2020). Minimizing blood loss in spine surgery. Glob. Spine J..

[12] He Y, Xu L, Ren L, Liu B (2019). Clinical analysis of complications of lumbar spinal stenosis in elderly patients with lumbar spinal stenosis. Hebei Med..

[13] Qi M, Wang S, Wang L, Chen X, Zhan W, Zhu X, Wang H (2022). Effects of oral and intravenous tranexamic acid on perioperative blood loss after lumbar spinal canal decompression and fusion. Chin. J. Orthopa. Trauma.

[14] Zhang Y, Wang X, Zhao Q, Shui C, Sun H, Hao D (2018). Effect of intravenous tranexamic acid on perioperative hidden blood loss in percutaneous pedicle screw fixation for thoracolumbar fractures. Chin. J. Orthop Trauma.

[15] Chang L, Xiong W, Liu HZ, Liu XY (2017). A clinical study on the topical application of tranexamic acid + gelatin sponge in lumbar surgery. Chin. J. Bone Joint.

[16] Cheriyan T, Maier SP, Bianco K, Slobodyanyuk K, Rattenni RN, Lafage V, Schwab FJ, Lonner BS, Errico TJ (2015). Efficacy of tranexamic acid on surgical bleeding in spine surgery: A meta-analysis. Spine J..

[17] Yu C, Kadri O, Kadado A, Buraimoh M, Pawloski J, Bartol S, Graziano G (2018). Intravenous and oral tranexamic acid are equivalent at reducing blood loss in thoracolumbar spinal fusion: A prospective randomized trial. Spine.

[18] Hsieh PW, Chen WY, Aljuffali IA, Chen CC, Fang JY (2013). Co-drug strategy for promoting skin targeting and minimizing the transdermal diffusion of hydroquinone and tranexamic acid. Curr. Med. Chem..

[19] Zhou Z, Huang Y, Yang H, Weng X, Li T, Wang G, Zhang Z, Liu T, Chen Y, Shen H (2019). Expert consensus on the application of tranexamic acid and anticoagulant for the enhanced recovery after orthopedic surgery in China. Chin. J. Bone Joint Surg..

[20] Zou Q (2022). Research progress of perioperative prophylactic anticoagulation therapy in lumbar spine fusion. Hainan Med. J..

[21] Chen Y, Zhao J, Yu Y, Liu X, Lin L, Zhang F, Linhardt RJ (2018). Antithrombin III-binding site analysis of low-molecular-weight heparin fractions. J. Pharm. Sci..

[22] Pannucci CJ, Fleming KI, Bertolaccini CB, Prazak AM, Huang LC, Pickron TB (2019). Assessment of anti-factor Xa levels of patients undergoing colorectal surgery given once-daily enoxaparin prophylaxis: A clinical study examining enoxaparin pharmacokinetics. JAMA Surg..

[23] Chen R, Luo H, Yang J (2021). Research progress on action mechanism and clinical application of low molecular weight heparin. Chin. Mod. Doct..

[24] Luo H, Yu G, Wang H, Zheng L, Yan W, Yao Q, Wang J, Xu X, Sun T, Zhang J (2020). A meta-analysis of rivaroxaban effectiveness and safety for the prevention of thromboembolism after total hip or knee arthroplasty. Chin. J. Bone Joint.

[25] Zhu D, Lu L, Fang B, Li Z (2019). Comparison efficacy and safety of two anticoagulants in preventing deep vein thrombosis after thoracolumbar fracture internal fixation. J. Clin. Orthop..

[26] Liu F, Wang J (2021). Research status on the mechanism and application of indobufen. Chin. J. Clin. Pharmacol..

[27] Patrono C, Baigent C, Hirsh J, Roth G (2008). Antiplatelet drugs: American college of chest physicians evidence-based clinical practice guidelines (8th edition). Chest.

[28] Wei J, Li W, Pei Y, Shen Y, Li J (2016). Clinical analysis of preoperative risk factors for the incidence of deep venous thromboembolism in patients undergoing posterior lumbar interbody fusion. J. Orthop. Surg. Res..

[29] Xu F, Yang P, Liu S, Zhen T, He Z, Li H (2018). Effect and safety of local medication of tranexamic acid on reducing postoperation blood loss inposterior lumbar fusion. Lingnan Mod. Clin. Surg..

[30] Tan X, Kuang R, Xie J, Zeng B, Xie J, Wei W, Wang H, Hu X, Chen L, Pi C (2017). Effects of tranexamic acid for spinal surgery during perioperative period: A systematic review and Meta-analysis. Chin. J. Traum..

